# Time Processing, Interoception, and Insula Activation: A Mini-Review on Clinical Disorders

**DOI:** 10.3389/fpsyg.2020.01893

**Published:** 2020-08-18

**Authors:** Carmelo Mario Vicario, Michael A. Nitsche, Mohammad A. Salehinejad, Laura Avanzino, Gabriella Martino

**Affiliations:** ^1^Dipartimento di Scienze Cognitive, Psicologiche, Pedagogiche e Degli Studi Culturali, Università di Messina, Messina, Italy; ^2^Department of Psychology and Neurosciences, Leibniz Research Centre for Working Environment and Human Factors, Dortmund, Germany; ^3^Department of Experimental Medicine, Section of Human Physiology and Centro Polifunzionale di Scienze Motorie, University of Genoa, Genoa, Italy; ^4^Department of Clinical and Experimental Medicine, University of Messina, Messina, Italy

**Keywords:** time processing, clinical disorders, interoception, insula, timing deficits

## Abstract

Time processing is a multifaceted skill crucial for managing different aspects of life. In the current work, we explored the relationship between interoception and time processing by examining research on clinical models. We investigated whether time processing deficits are associated with dysfunction of the interoceptive system and/or insular cortex activity, which is crucial in decoding internal body signaling. Furthermore, we explored whether insular activation predicts the subjective experience of time (i.e., the subjective duration of a target stimulus to be timed). Overall, our work suggests that alteration of the interoceptive system could be a common psychophysiological hallmark of mental disorders affected by time processing deficits.

## Introduction

The ability to track time is the result of a complex neural network, which includes a large set of cognitive and motor functions. A demonstration of the multidimensionality of timing ability is provided by the variety of terminologies associated with this mental function.

A classic distinction in the literature on time processing is made between prospective and retrospective timing (Block et al., 2018). The first involves advance knowledge of the duration of the target interval to be timed; the second implies that it is not known in advance that time of a target has to be estimated (Block et al., 2018).

A further element of time processing is the time scale of the phenomenon to be timed. In general, we distinguish between the ability to time sub-second and supra-second durations. These different temporal domains relate with the activity of segregated and relatively independent brain structures (see Ivry and Schlerf, 2008).

It is also important to distinguish between motor and perceptual timing. The first refers to the execution of a motor act to represent a temporal interval, as in the case of a time-line bisection task (e.g., Vicario, 2011), temporal production/reproduction (Vicario et al., 2011a, b), finger tapping (O’Regan et al., 2017), and movement synchronization (Vicario, 2012). The second involves a time comparison task, such as performing a task of discrimination between the duration of a reference and the duration of a target stimulus (e.g., Grondin et al., 1999; Vicario et al., 2009, 2012).

Finally, when it comes to timing, one could distinguish two dimensions, namely, time perception and time representation. The first refers to a synchronic (i.e., almost a real time) experience with the target to be timed. The second refers to diachronic (i.e., offline) experience with the target to be timed, that is, a timing that asks to make estimations about a target that was previously experienced or that is going to be experienced. With regard to time representation, making estimations about a target that was previously experienced requires a more direct involvement of memory processes, although it takes place also in the case of a target that is going to be experienced. In the second case, it is required to make predictions about the duration of the target to be timed, such as in the case of sports actions, where the accuracy in predicting the duration of a target (i.e., a moving ball) is crucial for successful sportive performance (Vicario et al., 2016).

The neural correlates of time processing include a wide range of cortical and subcortical structures, including the frontal and parietal cortex, the basal ganglia, the cerebellum, and the insula (for some reviews and discussion, see Ivry and Schlerf, 2008; Vicario and Martino, 2010; Vicario et al., 2013a, b; Bareš et al., 2019; Nani et al., 2019; Salehinejad et al., 2019, 2020). At the cognitive level, timing skills have been linked with attention, memory (Lewis and Miall, 2006), motor control (Avanzino et al., 2016), and interoception (Craig, 2009a; Meissner and Wittmann, 2011). Owing to this vast neural network, timing deficits are reported in various mental disorders (i.e., neurological and psychiatric), whose etiopathology involves the abnormal activity of at least one of the neuronal structures mentioned previously.

The evidence of an intricate neurocognitive network involved in time processing skills is a proof against the existence of a “mental clock” localized in a specific region of the brain. However, it makes it difficult to clarify if and what the majority (if not all) of mental disorders affected by timing deficits share at the psychophysiological level.

The goal of the current work is focusing on the contribution of interoception in the experience of time. Evidence in healthy humans (e.g., Pollatos et al., 2014b) shows that interoceptive awareness of the cardiac cycle is crucial to encode and reproduce durations of the range between 2 and 25 s (see also Meissner and Wittmann, 2011; Pollatos et al., 2014a). Moreover, we recently demonstrated that the manipulation of interoceptive signaling (i.e., appetite) affects the experience of time (Vicario et al., 2019). Finally, the insular cortex, a key neural region in decoding interoceptive signaling (Craig, 2009b), including the visceral emotion of disgust (see Vicario et al., 2017a, b for review) and appetite (Tataranni et al., 1999; Del Parigi et al., 2002), is implicated in the experience of time in healthy humans (e.g., Tregellas et al., 2006; Craig, 2009a; Wiener et al., 2010 for review). In keeping with this literature, we aim to explore the hypothesis that alterations in time processing, as reported in several clinical disorders, may be linked, at least in part, with dysfunctions of their interoceptive system, which depends on abnormal activity of insular cortex, as suggested by several works (e.g., Craig, 2009; Livneh et al., 2020). We have examined the available literature (giving priority to systematic reviews and/or meta-analysis) on timing deficits in clinical (i.e., neurological and psychiatric) disorders (schizophrenia, depression, anxiety and related disorders, eating disorders, attention-deficit hyperactivity disorder (ADHD), Tourette’s syndrome (TS), autism, Parkinson’s disease (PD), dystonia, essential tremor (ET), migraine) to explore the existence of dysfunctions of their interoceptive system at the behavioral and neural levels. Furthermore, we studied the existence of any specific relation between insular activation, which is involved in detecting/decoding interoceptive signaling (Craig, 2009b), and the subjective experience of time. In line with our aims, we only focused on research including supra-second durations, in keeping with the evidence of larger insula activation for this temporal range (Wittmann et al., 2010).

### Schizophrenia

The literature on timing skills in schizophrenia is quite extensive (for recent contributions, see Capa et al., 2014; Giersch et al., 2015; Martin et al., 2018; Wilquin et al., 2018). In the meta-analysis by Thoenes and Oberfeld (2017), the authors documented a small and task-dependent tendency of patients to overestimate durations in time estimation/production. Timing dysfunctions can span from millisecond to second durations (Carroll et al., 2009). A similar result is reported in a subsequent meta-analysis (Ueda et al., 2018) in which a relation between positive symptoms of schizophrenia and temporal overestimation of supra-second durations was described (Ueda et al., 2018). Finally, alterations in time processing have been documented also via qualitative approaches such as by using inductive summarizing content analysis (Vogel D. H. V. et al., 2019), suggesting a link between disturbances in the experience of time and alterations in the constitution of the stream of consciousness (Vogel D. H. V. et al., 2019).

Schizophrenia is also affected by altered interoceptive function. Ardizzi et al. (2016) demonstrated a reduced interoceptive accuracy (measured via the heartbeat perception task) in this clinical population. Interestingly, the authors reported also a positive relationship between interoceptive accuracy and the positive symptomatology in this clinical population. Moreover, reduced insula activation was reported during the execution of a time discrimination task (Davalos et al., 2011). Overall, the literature suggests alterations of timing, and dysfunctions at the level of insula, and one study directly relates these two observations. Because positive symptoms positively correlate with interoceptive accuracy (Ardizzi et al., 2016) and time overestimation performance (Ueda et al., 2018) in schizophrenia, we speculate that their interoceptive dysfunction might potentially play a direct role in their timing response (i.e., temporal overestimation). Talking about the potential link between insular activation and the perceived duration, the study by Davalos et al. (2011) does not provide insight in this regard, as the data analysis provided in this study focused on the percentage of errors, with no information on the direction of errors (i.e., overestimation or underestimation error).

### Depression

Depressed patients frequently report to perceive time as going by very slowly (Thönes and Oberfeld, 2015). As in the case of schizophrenia, also for depression further insights are provided by qualitative research (Vogel et al., 2018) documenting disturbances in the experience of time, specifically difficulties with respect to influencing or changing the present, resulting in an impersonal and blocked future (Vogel et al., 2018). Bschor et al. (2004) provided one of the first studies on time sense in depression and documented a uniform time overestimation pattern of supra-second durations in patients affected by major depressive episode and manic episode in time judgment tasks. This result has been confirmed in a more recent work using a time reproduction task (Mioni et al., 2016). A meta-analysis on timing skills in depression (see Thönes and Oberfeld, 2015) did not provide conclusive results. However, this might be a result of the inclusion of studies on patients under medications that might have played some independent effect on timing performance.

Depression is also characterized by altered interoceptive functions. It was suggested that this clinical population is affected by alliesthesia for internal body signals (Paulus and Stein, 2010), as well as abnormal body perception (increased interoceptive awareness) and altered (increased) insula activation (see Sliz and Hayley, 2012 for a review).

Overall, the literature shows both an alteration of time processing and interoceptive dysfunction. Further studies are required to establish whether a link exists between these dysfunctions.

### Anxiety and Related Disorders

Anxiety is another psychiatric disorder typically associated with abnormal time processing. Mioni et al. (2016) documented an under-reproduction of supra-second durations in this clinical population via time reproduction, which might indicate an overestimation in duration. Interestingly, an overestimation timing pattern was recently reported also in patients affected by post-traumatic stress disorder – PTSD (Vicario and Felmingham, 2018a).

Both anxiety and PTSD are also known to be associated with interoceptive dysfunctions (Martino G. et al., 2019). In anxiety, as well as in depression, symptoms of alliesthesia for internal body signals are present (Paulus and Stein, 2010). In PTSD, typical symptoms of interoceptive dysfunction include autonomic hypervigilance, depersonalization, and derealization (Glenn et al., 2017).

With regard to the insular cortex, higher activation in anxiety during affective tasks was reported (e.g., for a review see Paulus and Stein, 2010). The study by Simmons et al. (2009) reported a reduced insular activity in PTSD during the execution of affective tasks. However, a further work has reported higher activation in the right insula in this clinical population for affective stimuli (Bruce et al., 2012), which is also confirmed by earlier investigations (Shin et al., 1999).

Overall, the literature shows both an alteration of time processing and interoceptive dysfunction. Further studies are required to establish whether a link exists between these dysfunctions.

### Eating Disorders

Patients with anorexia nervosa (AN) and hyperphagia are known for their deficit in perceiving interoceptive signaling. In AN, hunger insensitivity, food anxiety, and gastrointestinal complaints are reported (Khalsa et al., 2018), whereas hyperphagia symptoms include hypersensitivity to interoceptive signals of hunger and the inability to accurately detect interoceptive signals of satiety (Simmons and DeVille, 2017).

A dysfunction of the insular cortex is another hallmark in AN (Nunn et al., 2011) and might explain the high disgust sensitivity in this clinical population (Vicario, 2013a). A reduced BOLD response in the insula of recovered AN is reported in response to sucrose or water administration, compared with healthy controls (Wagner et al., 2008). In contrast, Scharmüller et al. (2012) found higher insular activation in response to food cues for obese people (for a review, see Frank et al., 2013).

In terms of time-keeping skills in eating disorders, two recent contributions document a temporal underestimation in AN (Vicario and Felmingham, 2018b) and a temporal overestimation in obesity (Vicario et al., 2019) for the estimation of supra-second durations.

Overall, the literature provides preliminary evidence of both an alteration of time processing and interoceptive dysfunction. Further work is required to establish whether a link exists between these dysfunctions.

### Attention-Deficit Hyperactivity Disorder

The literature on timing deficits in ADHD is well consolidated (Toplak et al., 2006). Mullins et al. (2005) reported underestimation of supra-second durations in a time reproduction task (see also Pollak et al., 2009; Noreika et al., 2013, for a review). An fMRI study (Valera et al., 2010) found decreased insular activation during the execution of a sub-second timing – sensorimotor synchronization – task in ADHD. This pattern was confirmed in a meta-analysis of fMRI studies of timing in ADHD (Hart et al., 2012). Finally, a recent study by Wiersema and Godefroid (2018) did not reveal a significant difference between patients and healthy controls in the execution of interoceptive tasks, suggesting preserved interoceptive awareness in this clinical population. Overall, the research examined previously does not provide support to the hypothesis of a linking between interoception and timing deficits in ADHD.

### Tourette’s Syndrome

The research on timing skills in TS is still in its infancy. Studies of Vicario et al. (2010, 2016) confirmed a tendency of this clinical population to overestimate supra-second durations, in the absence of pharmacological treatment, in a time reproduction task (see also Martino D. et al., 2019; Vicario et al., 2020, for further contributions in the field of time processing). This response pattern correlated with tic severity (Vicario et al., 2010). Interestingly, fMRI research has shown an association between premonitory urges (tic severity) and the involvement of higher insular activation (for a review, see Worbe et al., 2015; Cavanna et al., 2017) in this clinical population. Pile et al. (2018) also confirmed reduced interoceptive accuracy, compared with healthy controls. Moreover, Ganos et al. (2015) found a positive correlation between premonitory urges (tic severity) and interoceptive awareness. Given the evidence that tic severity relates with time overestimation, interoceptive awareness, and insular activation, we speculate that this timing pattern can be potentially linked with higher activity of the insular cortex in TS.

Overall, the literature shows both an alteration of time processing and interoceptive dysfunction. Further studies are required to establish whether a link exists between these dysfunctions.

### Autism

Timing skills are abnormal in autism (e.g., for a review see Allman and Meck 2012; Falter et al., 2012). Temporal underestimation for supra-second durations has been observed in autism via time reproduction task (Martin et al., 2010). Moreover, by using an inductive content analysis, the results by Vogel D. et al. (2019) suggest that this disorder is affected by an interrupted time experience syndrome. A meta-analysis (Di Martino et al., 2009) documents right insula cortex hypoactivation in autism spectrum disorders during the execution of several affective/social tasks. In accordance, there is evidence of altered interoceptive functions in this clinical population (for a review, see Quattrocki and Friston, 2014; Garfinkel et al., 2016; Mulcahy et al., 2019). Overall, the research examined previously suggests that the time underestimation pattern in autism may be potentially linked with a lower activity of the insular cortex.

### Parkinson’s Disease

The literature on time processing in PD is wide (for a review, see Avanzino et al., 2016) and shows a relevance of dopaminergic degeneration/dysfunctions for this cognitive function (Lewis and Miall, 2006; Vicario, 2013b). The timing pattern most frequently reported in these patients in a tendency to underestimate supra-second durations in the absence of dopaminergic therapy (e.g., Koch et al., 2008) in a time reproduction task. Interestingly, the study by Harrington et al. (2011) in PD (L-Dopa off-state therapy) documented a reduction of insular activation during encoding of supra-second durations in these patients. Finally, evidence does exist for interoceptive alterations in PD. Ricciardi et al. (2016) documented lower interoceptive sensitivity (measured via the heartbeat perception task) in PD, as compared with controls. Overall, the literature shows both an alteration of time processing and interoceptive dysfunction. More research is required to establish whether a link exists between these dysfunctions.

### Dystonia

The behavioral research in dystonia has documented timing deficits in cervical dystonia (Martino et al., 2015), as well as in writer’s cramp (Avanzino et al., 2013). Both studies found an enhanced absolute value of timing error during the execution of a temporal expectation task, possibly linked with a cerebellar dysfunction (Avanzino et al., 2015; Martino et al., 2020), whereas no difference was reported with regard to the direction of timing performance (i.e., patients and controls underestimated longer durations in similar way). Ferrazzano et al. (2017) have recently documented reduced interoceptive sensitivity, as measured via the heartbeat detection task, in cervical dystonia. Moreover, there is evidence for reduced insular activity in this clinical population (Opavský et al., 2012). A direct link between time alteration and interoceptive dysfunction in this disease remains to be established.

Overall, the literature shows an alteration of time processing with no evidence of interoceptive dysfunction. More research is required to establish whether a link exists between these dysfunctions.

### Essential Tremor

No published research has explored interoceptive sensitivity in ET so far. Nevertheless, a recent neuroimaging work has reported a decreased amplitude of low-frequency fluctuations (ALFFs) of blood oxygen level–dependent signals in correspondence of insular cortex (Wang et al., 2018). ALFF is an index used to characterize regional cerebral function (Wang et al., 2018). One study (Pedrosa et al., 2016) documents furthermore temporal underestimation in a reproduction task for both sub-second and supra-second durations in ET.

Overall, the literature shows an alteration of time processing with no evidence of interoceptive dysfunction. More research is required to establish whether a link exists between these dysfunctions.

### Migraine

The literature on time processing in migraine offers some contribution. In a first investigation (Anagnostou and Mitsikostas, 2005), a timing overestimation of sub-second durations was documented in adults affected by migraine and depression. This pattern was replicated in a subsequent work (Zhang et al., 2012) in the absence of depression. More recently, a time overestimation response of supra-second durations was reported in a sample of adolescents affected by migraine (Vicario et al., 2014). In a recent review, Borsook et al. (2016) linked migraine with structural and functional alterations of insula. For example, Xue et al. (2012) reported increased insular activity during the resting-state fMRI (Xue et al., 2012). Moreover, migraine can be intended as an example of interoceptive disorder for the interoceptive nature of some associated symptoms (e.g., nausea, vomiting) (see Brennan and Pietrobon, 2018 for a review). Overall, the literature shows both an alteration of time processing and interoceptive dysfunction. More research is required to establish whether a link exists between these dysfunctions.

## Discussion

In this work, we explored if alterations of interoceptive functions and/or dysfunctions of the insular cortex can be found in clinical disorders affected by timing deficits. Overall, the examined research provides preliminary support to this hypothesis, although we found only three papers that directly relate timing and the insula.

With regard to the relevance of insular activation to predict the direction (overestimation vs. underestimation) of timing performance, the overall picture is not consistent. In the majority of the examined clinical disorders, the literature shows, in independent contributions, time underestimation and reduced insula activation (i.e., PD, ET, autism, ADHD), and time overestimation and increased insula activation (i.e., TS, schizophrenia, depression, anxiety, and migraine). Interestingly, the latter timing pattern is in line with the study of Dirnberger et al. (2012) in healthy humans, documenting greater activation of the insula when time is overestimated. Moreover, this latter pattern might be associated with PTSD, if we focus on evidence of a larger insula activation.

In one third of the examined clinical disorders, the literature shows, again in independent contributions, time overestimation and reduced insula activation (i.e., hyperphagia) or time underestimation and increased insula activation (i.e., anxiety and AN). In dystonia, the results are not conclusive.

The role of insula on time processing seems to be selectively related with specific timing tasks, a specific stage of time processing and a specific duration range (i.e., supra-second duration) (e.g., the study of Wittmann et al., 2010 shows higher insula activation in the encoding stage of supra-second durations during a time reproduction). This suggests that the ideal task to test the relevance of interoceptive/insular cortex function on timing skills of the examined clinical populations should include these features. In keeping with these premises, if we focus our analysis on clinical populations (i.e., TS, migraine, ET, PD, autism, ADHD, depression, anxiety) tested via time reproduction of supra-second durations, the link between insula activity and the direction of the timing response appears consistent in all cases. A match (of independent reports) between increased insular activity and time overestimation is reported in migraine, TS, depression, and anxiety; vice versa, a match between decreased insular activity and time underestimation is reported in ET, PD, autism, and ADHD.

Limitations, which need to be addressed in future research, include various aspects. The current work does not allow providing a direct demonstration that timing performance in the examined clinical populations is linked with their interoceptive alterations as, in most cases, the examined literature has not directly explored this issue. Therefore, the analysis provided in our work should be taken with caution and considered as a starting point for a more systematic investigation of the topic addressed in our article. Furthermore, the number of neuroimaging investigations exploring timing skills in clinical populations is low: neuroimaging was included only in three clinical populations, and none of these three studies included a supra-second time reproduction task. Another limitation is the involvement of heterogeneous clinical populations with regard to pharmacological treatment. This might have contributed to inconsistent results, as several of the examined mental disorders are treated with dopaminergic and/or serotonergic drugs, which are known to influence temporal performance (Rammsayer, 1993). Finally, the heterogeneity of tasks/procedures adopted to test timing skills in their clinical populations should be taken into account.

The adoption of time processing protocols more directly related with the activity of insula and/or interoceptive functions (e.g., judging the timing of own heartbeats – Critchley et al., 2004), in combination with neuroimaging investigations and measures of interoceptive sensitivity, will allow to provide a direct contribution to the current hypothesis and, therefore, address the limits of our analysis, which are bounded with the limits of the current state of the art.

In conclusion, our work provides preliminary evidence in support of the hypothesis that insular cortex alterations, which probably play a main role in interoceptive dysfunctions of the examined clinical disorders, may contribute to explain timing deficits. Our results also provide preliminary evidence that the insula activity predicts the direction (over/underestimation) of the experience of time, when measured via supra-second time reproduction tasks. The exploration of the connection between insula activity, interoceptive dysfunctions, and timing alterations is a timely topic as it would contribute to expand the current knowledge/debate about how the gut–brain interaction influences cognitive and affective processes.

## Author Contributions

CV made significant contribution to the design of the review, the drafting, and revision of the manuscript. MN gave a significant contribution to the drafting and the revision of the manuscript. MS provided substantial contribution in the revision of the manuscript. LA provided substantial contribution in the drafting and revision of the manuscript. GM critically revised the manuscript and gave the final approval of the manuscript to be submitted. All authors contributed to the article and approved the submitted version.

## Conflict of Interest

The authors declare that the research was conducted in the absence of any commercial or financial relationships that could be construed as a potential conflict of interest.
